# Alcohol consumption and associations with sociodemographic and health-related characteristics in Germany: A population survey

**DOI:** 10.1016/j.addbeh.2021.107159

**Published:** 2022-02

**Authors:** Claire Garnett, Sabrina Kastaun, Jamie Brown, Daniel Kotz

**Affiliations:** a Department of Behavioural Science and Health, University College London, London, UK; b SPECTRUM Consortium, London, UK; c Institute of General Practice, Addiction Research and Clinical Epidemiology Unit, Centre for Health and Society, Medical Faculty of the Heinrich-Heine-University, Düsseldorf, Germany

**Keywords:** Alcohol consumption, Prevalence, Germany, Adults, Hazardous drinking, Population survey, AUDIT-C, Alcohol Use Disorders Identification Test – Consumption, AUDIT, Alcohol Use Disorders Identification Test, CI, Confidence interval, DEBRA, Deutsche Befragung zum Rauchverhalten, DEGS, German Health Interview and Examination Survey for Adults, ESA, Epidemiological Survey of Substance Abuse, GEDA, German Health Update, PHQ-4, Patient Health Questionnaire-4, SD, Standard deviation, SEP, Socioeconomic position

## Abstract

**Background:**

To assess the prevalence of ever-drinking and hazardous drinking among adults in Germany, and investigate the factors associated with level of alcohol consumption.

**Methods:**

Cross-sectional population survey of a representative sample of 11,331 adults in Germany (2018 to 2019). The Alcohol Use Disorders Identification Test-Consumption (AUDIT-C) was used to define ever-drinking (AUDIT-C>=1), hazardous drinking (AUDIT-C>=5) and an overall AUDIT-C (alcohol consumption) score (from 0 to 12). Regression models were used to examine sociodemographic and health-related characteristics associated with AUDIT-C score.

**Results:**

The prevalence of ever-drinking and hazardous drinking was 84.7% (95% CI = 84.1–85.4) and 19.4% (95% CI = 18.6–20.1), respectively. The mean AUDIT-C score was 2.8 (SD = 2.16). AUDIT-C scores were independently positively associated with having medium (B_adj_ = 0.12, 95% CI = 0.02–0.21) and high (B_adj_ = 0.11, 95% CI = 0.01–0.21) educational qualifications (compared with low), monthly income (B_adj_ = 0.31 per €1,000, 95% CI = 0.26–0.36), being a current smoker (B_adj_ = 0.94, 95% CI = 0.86–1.02), anxiety (B_adj_ = 0.26, 95% CI = 0.02–0.50), and living in North East (B_adj_ = 0.43, 95% CI = 0.29–0.58), North West (B_adj_ = 0.47, 95% CI = 0.39–0.55) and South East (B_adj_ = 0.79, 95% CI = 0.64–0.93) Germany (compared with South West), and negatively associated with age (B_adj_ = -0.17, 95% CI = -0.21- −0.13), being female (B_adj_ = -1.21, 95% CI = -1.28- −1.14) and depression (B_adj_ = -0.22, 95% CI = -0.43- −0.02).

**Conclusion:**

In a large, representative sample of adults in Germany, the majority were ever-drinkers and one fifth were hazardous drinkers. Higher alcohol consumption scores were associated with being younger, male, current smoker, of high socioeconomic position, anxiety, and not living in South West Germany, and lower scores were associated with depression. These groups may benefit from targeted alcohol reduction policies and support.

## Highlights

• Prevalence of hazardous drinking was 19.4% among adults in Germany between 2018 and 2019.
• Heavier levels of alcohol consumption were associated with being younger and male.
• Heavier levels of alcohol consumption were associated with having anxiety.
• Lower levels of alcohol consumption were associated with depression.
• Certain groups may benefit from targeted alcohol reduction policies and support.

## 1 Introduction

Alcohol consumption accounts for 3.8% of deaths globally and 4.6% of global disability-adjusted life-years (Rehm et al., 2009). There is a dose–response relationship between alcohol consumption and alcohol-related harms (Alcohol and Public Policy Group, 2010, Fullman et al., 2018, Holmes et al., 2016). The risk of mortality rises with increasing levels of alcohol consumption, and the level of consumption that minimises health loss is zero (Fullman et al., 2018). There are also thresholds of drinking – often labelled hazardous drinking (i.e. increasing and higher risk drinking) and operationalised in terms of grams of alcohol consumed per week or scores on the Alcohol Use Disorders Identification Test (AUDIT) – which are judged to pose significant risk of harm to physical or mental health, or social consequences (National Institute for Health and Care Excellence, 2010). Ever-drinking can be defined as an AUDIT score of one or more (where an AUDIT score of zero indicates never drinking). Thresholds can be useful from an individual-level perspective, providing people with clear guidelines for their own behaviour. The dose–response relationship between consumption and harms means that estimates of overall consumption are necessary to accurately model current population-level impacts of alcohol.

Alcohol consumption varies between different countries, population subgroups, and over time (Alcohol and Public Policy Group, 2010). Europe has some of the highest per capita consumption levels in the world and the highest proportion of alcohol-attributable deaths (6.5%) (Rehm et al., 2009, World Health Organisation, 2014). In 2016, the annual alcohol per capita consumption in Germany (13.4 L of pure alcohol) was estimated to be higher than the average for the WHO European region (9.8 L), which in turn is higher than the global average (6.4 L) (World Health Organisation, 2018). It is critically important to have current national estimates on alcohol consumption to understand country-specific alcohol-related harms in Germany. There are three large, national population-based surveys which collected data on alcohol consumption in Germany: the Epidemiological Survey of Substance Abuse (ESA) (Atzendorf, Rauschert, Seitz, Lochbühler, & Kraus, 2019), the German Health Update (GEDA) (Lange et al., 2015), and the German Health Interview and Examination Survey for Adults (DEGS) (Lange, Manz, Rommel, Schienkiewitz, & Mensink, 2016). The most recent data for Germany comes from the ESA, which showed that the prevalence of hazardous drinkers (defined as more than 12/24 g/day for women/men on average) was 18.1% in 2018 (Atzendorf et al., 2019). Consumer data of per capita consumption (Statista, 2020) and population-based surveys have shown decreasing trends in alcohol consumption in Germany over time (Lange et al., 2016, Seitz et al., 2019). Therefore, it is important to update these findings using data from a representative sample across the whole of Germany.

It is also important to understand the factors associated with alcohol consumption so that targeted support can be provided. Previous research found that rates of hazardous drinking were higher among men (Atzendorf et al., 2020, Bott et al., 2005, Halme et al., 2008, Lange, Mankertz, & Kuntz, 2017, Lange et al., 2016) and those who were divorced or separated (Halme et al., 2008), and that hazardous drinkers were older than those who abstained or were moderate drinkers (Bott et al., 2005) with the highest prevalence in Germany among those aged 45–64 years (Lange et al., 2017). Higher socioeconomic position (SEP) was positively associated with alcohol consumption across different age groups in a cohort study (Pabst, van der Auwera, Piontek, Baumeister, & Kraus, 2019), and those with higher and intermediate education levels were more likely to report at-risk alcohol consumption in Germany (Atzendorf et al., 2020). The association between alcohol consumption and SEP has important implications for health-related inequality at the individual and country level (Grittner, Kuntsche, Gmel, & Bloomfield, 2013); however this association is complex (Boyd et al., 2021) with moderating factors, such as ethnicity and gender (Collins, 2016), and drinking patterns, such as heavy episodic drinking, contributing to socioeconomic inequalities in alcohol-related mortality (Probst, Kilian, Sanchez, Lange, & Rehm, 2020). Hazardous drinking is also independently associated with an increased risk of having an affective or anxiety disorder compared with abstinent or moderate drinkers (Bott et al., 2005). Furthermore, there is a positive association between hazardous drinking and smoking cigarettes (Falk et al., 2006, Hasin et al., 2007, Lange et al., 2016).

In addition, the geographic region of Germany appears to be related to prevalence of hazardous drinking (Atzendorf et al., 2020) with higher prevelance rates in East (18.3%) compared with West (14.6%) Germany, and in South (16.7%) compared with North (13.9%) Germany (Atzendorf et al., 2020). The federal states still vary by sociodemographic and macro-economic characteristics, with reported differences in terms of economic power remaining between East and West Germany (Bundesministerium für Wirtschaft und Energie. Jahresbericht der Bundesregierung zum Stand der Deutschen Einheit, 2017). There is also expected to be a South-North gradient based on the industrialisation with the Southern federal states showing a higher economic growth (WeltWirtschaftsInstitut, 2016) and lower unemployment rates (Bundesamt, 2017). Furthermore, there has been a decreasing trend in alcohol use in Germany in both men and women from 1995 to 2018, though the prevalence of episodic drinking showed an increasing trend in women but a decreasing trend in men (Seitz et al., 2019). This indicates the importance of adjusting for when a survey was conducted. Nevertheless, Germany is still one of the countries where the most alcohol is drunk (Peacock et al., 2018).

There is a need for an up-to-date study assessing the broad range of factors associated with alcohol consumption among adults in a representative sample across Germany. This study answered the following research questions among a representative sample of adults (aged 18 and over) across Germany between 2018 and 2019:

1. What is the prevalence of ever-drinking and hazardous drinking among adults in Germany and by federal state?
2. What is the average alcohol consumption (AUDIT-C) score and weekly alcohol consumption (in grams of alcohol) among adults in Germany and by federal state?
3. To what extent are sociodemographic characteristics, region of Germany, smoking status, symptoms of depression or anxiety among adults, or survey wave in Germany associated with:
  a. alcohol consumption (AUDIT-C) score, and
  b. prevalence of hazardous drinking?

## 2 Methods

### 2.1 Study design and population

We used data from the German Study on Tobacco Use (DEBRA: “Deutsche Befragung zum Rauchverhalten”, www.debra-study.info): an ongoing representative household survey on tobacco use in Germany, to which questions on alcohol consumption were added in 2018 (Kastaun et al., 2017). The DEBRA study collects bimonthly data from computer-assisted face-to-face household interviews of people aged 14+ years. The survey is conducted by a market research institute (Kantar). Respondents are selected wave-by-wave using a multi-stage, multi-stratified random probability sampling method (Supplementary File 1) and interviewed regarding their sociodemographic characteristics, tobacco and e-cigarette use, and alcohol consumption. In brief, after dividing the federal territory of Germany geographically into 53,000 small areas of approximately equal size, measuring points are generated per unit and household addresses are determined randomly. The interviewer then carries out a random walk in their area and choose the household determined according to a random walk procedure. Within this household, the target person is selected using a random process (“Schwedenschlüssel”), which gives an equal chance of selection to every eligible person (aged 14+ years) within a household (Kish, 1949). Further details on the methodology, sample selection and weighting technique have been published in a study protocol (Kastaun et al., 2017), and further materials are available on the Open Science Framework (https://osf.io/ndu6r/).

Data on alcohol consumption were collected across six waves between June/July 2018 and April/May 2019, with each wave comprising approximately 2,000 respondents. This study aggregated the data across these six waves from responders who were aged 18+ years. We focused on this group as 18 is the national legal age of sale in Germany for all alcohol (16 is the legal age for wine and beer only) and drinking trends among adolescents differ from adults (Pennay et al., 2018). Research questions 1 and 2 were repeated for respondents aged 14 to 17 (inclusive) and reported in supplementary materials.

The DEBRA study is registered on the German Clinical Trials Register (registration numbers DRKS00011322 and DRKS00017157) and has received ethical approval (HHU 5386R).

### 2.2 Measures

#### 2.2.1 Outcome measures

Three outcome measures were used: i) prevalence of ever-drinking, ii) prevalence of hazardous drinking, and iii) alcohol consumption score (continuous). All of these outcome measures were derived from the AUDIT-C, a three-item measure of alcohol consumption which includes questions on frequency, quantity, and frequency of binge drinking (Bradley et al., 2007, Reinert and Allen, 2007) (Supplementary File 1). The AUDIT-C score provides an alcohol consumption score and ranges from 0 to 12 (Bradley et al., 2007, Bush et al., 1998, Reinert and Allen, 2007). An AUDIT-C score of 0 indicates never drinking, and therefore ever-drinking was defined as an AUDIT-C score of 1 or more. An AUDIT-C score of 5 or more was used to indicate hazardous drinking (Rumpf et al., 2002, National Institute for Health and Care, 2018). The same cut-off for both men and women was used (as pre-specified in the study protocol), based on a study validating the AUDIT-C among the general population in Germany (Rumpf et al., 2002) and the National Institute for Health and Care Excellence guidance (National Institute for Health and Care, 2018). The AUDIT-C was also transformed into an estimate of weekly alcohol consumption (in grams of alcohol) to provide a more interpretable measure (Supplementary File 1). It was estimated from the AUDIT-C, as the product of frequency (item 1) by quantity (item 2) with adjustment for occasional heavy drinking (item 3) (Dutey-Magni, Brown, Holmes, & Sinclair, 2021).

#### 2.2.2 Predictor variables

Sociodemographic characteristics measured were: age (in years); sex (male versus female); marital status (married versus not [single, divorced, widowed]); region of Germany; and SEP was operationalised using two variables: educational qualifications (low [junior high school equivalent or no qualification], middle [secondary school equivalent], high [high school equivalent or advanced technical college equivalent]), and monthly net household income (continuous, range from 0 [€0 income] to 7 [€7,000 or more], see Supplementary File 1). The monthly net household income variable was dichtomised (based on the mean score) for analyses where prevalence estimates and mean level of alcohol consumption was stratified by predictor variables or by region and federal state.

Two variables were generated for region of Germany: i) the 16 German federal states for unadjusted models, with North Rhine-Westphalia as the reference group, since it is the most populous federal state, and ii) intermediate directions for adjusted models (using the groupings from a previous study (Atzendorf et al., 2020) – South-West (reference group; Baden-Wuerttemberg, Bavaria, Hesse, Rhineland-Palatinate, Saarland), North-East (Brandenburg, Mecklenburg-Western Pomerania, Saxony-Anhalt), North-West (Berlin, Bremen, Hamburg, Lower Saxony, North Rhine-Westphalia, Schleswig-Holstein), and South-East (Saxony, Thuringia). A separation in West and East Berlin is not possible so Berlin was coded as North-West with sensitivity analyses conducted where it was coded as North-East.

Other health-related characteristics measured were: smoking status (current smoker versus not [ex-smoker and never smoker]), depression, and anxiety. Depression and anxiety were measured using the validated Patient Health Questionnaire-4 (PHQ*-*4) with scores of 3 or above across the depression and anxiety subscales indicating probable cases of depression and anxiety, respectively (Kroenke et al., 2009, Löwe et al., 2010) (see Supplementary File 1 for details). The questions on depression and anxiety were optional and 10.9% (n = 1,240) and 11.0% (n = 1,242) of respondents, respectively, declined to answer these questions.

#### 2.2.3 Potential confounding variables

We adjusted for study wave to account for temporal trends in alcohol consumption (de Vocht et al., 2016) with study wave coded as a categorical variable (June to September 2018 [reference group]/October 2018 to January 2019/February to May 2019).

### 2.3 Statistical analyses

The analysis plan was pre-registered on Open Science Framework (https://osf.io/h5y6m/). Data were weighted to be representative of the German population accounting for personal and household characteristics, and the weighted data were used for research questions 1 and 2. In line with the multi-stage sampling procedure, the weighting was conducted in separate stages to differentiate between the design weighting (which corrects unequal selection probabilities due to sample design and is calculated by an analytical approach) and the outcome weighting (which reweights cases who actually participated in the survey compared with known general population parameters and is calculated as rim-weighting within an iterative process). Further details on the weighting technique are described in the study protocol (Kastaun et al., 2017). The regression analyses for research question 3 used unweighted data.

As the questions on depression and anxiety were optional, complete case analysis was conducted for all other variables of interest using SPSS. Multiple imputation was used to impute the missing values for the depression and anxiety variables using all other variables as predictors. Five imputed data sets were created (Graham, Olchowski, & Gilreath, 2007), analysed individually and then the results were combined using Rubin's rules (Rubin, 2004), to produce the reported pooled estimates. A sensitivity analysis was conducted for complete cases of all variables of interest, including depression and anxiety.

#### 2.3.1 Prevalence of ever-drinking and hazardous drinking

**Fig. 1:**
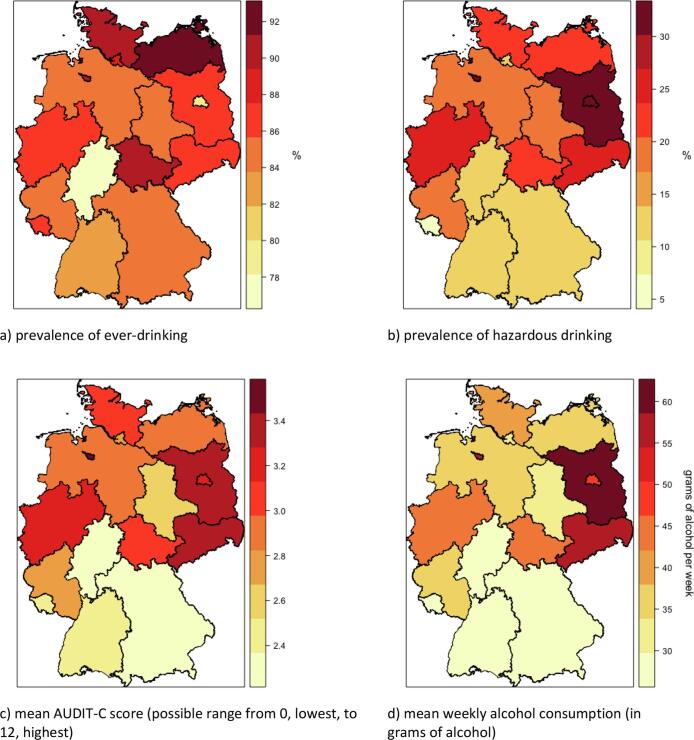
Map of Germany showing the prevalence of a) ever-drinking and b) hazardous drinking and c) mean alcohol consumption score and d) weekly alcohol consumption in each federal state. Figures created in RStudio v1.1.414.

We report the proportion (and 95% confidence interval [CI]) of the general population of adults who were ever-drinkers and hazardous drinkers. The prevalence estimates were then stratified by predictor variables and each federal state. See Fig. 1 for a map of Germany showing the prevalence of ever-drinking and hazardous drinking in each federal state.

#### 2.3.2 Average alcohol consumption (AUDIT-C) score and weekly alcohol consumption

We report the mean (and standard deviation [SD]) alcohol consumption (AUDIT-C) score and weekly alcohol consumption (in grams of alcohol), and present this stratified by predictor variables and each federal state. See Fig. 1 for a map of Germany showing the mean alcohol consumption score and weekly alcohol consumption in each federal state.

#### 2.3.3 Factors associated with alcohol consumption (AUDIT-C) score and prevalence of hazardous drinking

Linear and logistic regression models were used to analyse unadjusted and adjusted associations of AUDIT-C score and prevalence of hazardous drinking, respectively, with age, sex, marital status, educational qualifications, monthly income, region of Germany, smoking status, depression, anxiety, and survey wave. Age was transformed (by dividing the original variable by the SD) for ease of interpretation of model coefficients. We ran sensitivity analyses using the transformed weekly alcohol consumption measure.

In the unadjusted models, all 16 federal states were entered individually for region of Germany with North Rhine-Westphalia as the reference. In the adjusted models, region of Germany was entered as South-West (reference), North-East, North-West and South-East. Sensitivity analyses were conducted where Berlin was coded as North-East.

## 3 Results

### 3.1 Sample characteristics

A total of 11,331 adults aged 18+ years in the general population in Germany participated in the study between June/July 2018 and April/May 2019 with complete data for all variables of interest except for depression and anxiety. Sample characteristics are reported in Table 1 for all respondents (weighted n = 11,127) and for those who had complete cases for all predictor variables, including depression and anxiety measures (n = 10,069, weighted n = 9,937).

**Table 1:** Sample characteristics.

		All respondents (n = 11,127)^a^	Respondents with complete cases for anxiety and depression (n = 9,937)
Age, mean (SD)		51.5 (18.43)	51.5 (18.42)
Sex, % female (n)		52.0 (5,785)	52.2 (5,192)
Marital status, % married (n)		57.1 (6,357)	56.6 (5,625)
*Educational qualifications*			
	% low (n)	30.5 (3,397)	30.8 (3,062)
	% medium (n)	37.9 (4,216)	38.3 (3,808)
	% high (n)	31.6 (3,514)	30.9 (3,067)
Monthly income score^b^, mean (SD)		1.7 (0.82)	1.7 (0.82)
Smoking status, % current smoker (n)		29.9 (3,327)	30.3 (3,013)
Depression^c^, % yes (n)		4.6 (5 1 4)	4.5 (4 4 6)
Anxiety^d^, % yes (n)		3.1 (3 4 5)	3.0 (2 9 7)

Data weighted to be representative of the German population

a Complete cases for all variables of interest except for depression and anxiety where multiple imputation was used

b Range from 0 (€0 income) to 7 (€7,000 or more)

c A score of 3 or more on the depression subscale of the PHQ-4

d A score of 3 or more on the anxiety subscale of the PHQ-4

### 3.2 Prevalence of ever-drinking and hazardous drinking

Among adults in the general population of Germany, 84.7% (95% CI = 84.1, 85.4) were ever-drinkers and 19.4% (95% CI = 18.6, 20.1) were hazardous drinkers. The prevalence estimates stratified by the predictor variables are presented in Table 2, and by each federal state and region are presented in Table 3. The sensitivity analysis with complete cases of all variables (including depression and anxiety; n = 9,937) showed a similar pattern of results (Supplementary Table 1 and Supplementary Table 2).

**Table 2:** Ever-drinker and hazardous drinker prevalence estimates, and mean AUDIT-C score and weekly alcohol consumption among adults in Germany, stratified by predictor variables.

		Prevalence of ever-drinkers, % (95% CI)	Prevalence of hazardous drinkers, % (95% CI)	AUDIT-C score, mean (SD)	Weekly alcohol consumption in grams, mean (SD)
All respondents (n = 11,127)		84.7 (84.1, 85.4)	19.4 (18.6, 201)	2.8 (2.16)	37.9 (56.43)
*Age*					
	18–24 (n = 1,054)	83.7 (81.5, 86.0)	24.7 (22.1, 27.3)	3.1 (2.43)	40.0 (60.66)
	25–34 (n = 1,380)	87.9 (86.2, 89.6)	21.3 (19.1, 23.4)	2.9 (2.21)	38.2 (61.67)
	35–44 (n = 1,623)	85.3 (83.5, 87.0)	21.5 (19.5, 23.5)	2.9 (2.17)	36.9 (49.71)
	45–54 (n = 2,048)	88.2 (86.8, 89.6)	21.3 (19.5, 23.1)	2.9 (2.05)	39.3 (54.02)
	55–64 (n = 1,958)	88.1 (86.7, 89.6)	21.7 (19.9, 23.5)	3.1 (2.21)	45.5 (67.27)
	65+ (n = 3,065)	78.9 (77.5, 80.4)	12.7 (11.5, 13.9)	2.3 (1.98)	31.8 (48.59)
*Sex*					
	Male (n = 5,343)	89.2 (88.4, 90.0)	29.6 (28.4, 30.8)	3.5 (2.32)	52.5 (69.78)
	Female (n = 5,785)	80.6 (79.6, 81.6)	9.9 (9.1, 10.7)	2.1 (1.78)	24.5 (35.39)
*Marital status*					
	Not (n = 4,770)	82.8 (81.2, 83.9)	21.1 (19.9, 22.2)	2.8 (2.30)	38.0 (58.74)
	Married (n = 6,357)	86.2 (85.3, 87.0)	18.1 (17.1, 19.0)	2.8 (2.05)	37.8 (54.64)
*Educational qualifications*					
	Low (n = 3,397)	79.5 (78.2, 80.9)	17.9 (16.6, 19.2)	2.5 (2.25)	36.4 (62.62)
	Medium (n = 4,216)	87.1 (86.1, 88.1)	21.3 (20.1, 22.6)	2.9 (2.19)	40.13 (58.31)
	High (n = 3,514)	87.0 (85.8, 88.1)	18.4 (17.2, 19.7)	2.9 (2.00)	36.7 (46.97)
*Income*^a^					
	Low (n = 6,632)	81.7 (80.8, 82.7)	17.6 (16.7, 18.5)	2.6 (2.16)	34.7 (55.51)
	High (n = 4,495)	89.2 (88.3, 90.1)	22.0 (20.8, 23.2)	3.1 (2.12)	42.7 (57.43)
*Smoking status*					
	Never or ex-smoker (n = 7,801)	83.3 (82.5, 84.1)	13.5 (12.8, 14.3)	2.5 (1.92)	30.7 (44.02)
	Current smoker (n = 3,327)	88.2 (87.1, 89.3)	33.0 (31.4, 34.6)	3.5 (2.48)	54.7 (75.53)
*Depression*^d^					
	No (n = 10,614)	85.5 (84.8, 86.2)	19.4 (18.6, 20.1)	2.8 (2.14)	37.8 (55.48)
	Yes^b^ (n = 514)	69.5 (64.1, 74.9)	18.5 (14.8, 22.2)	2.5 (2.56)	39.6 (73.19)
*Anxiety*^d^					
	No (n = 10,783)	85.1 (84.5, 85.8)	19.3 (18.6, 20.0)	2.8 (2.13)	37.5 (54.64)
	Yes^c^ (n = 345)	72.2 (67.2, 77.2)	21.0 (16.3, 25.7)	2.8 (2.95)	50.4 (96.15)

Data weighted to be representative of the German population

Complete cases for all variables of interest except for depression and anxiety where multiple imputation was used

a Dichotomised based on the mean score of 1.69 (range from 0 [€0 income] to 7 [€7,000 or more])

b A score of 3 or more on the depression subscale of the PHQ-4

c A score of 3 or more on the anxiety subscale of the PHQ-4

d SD calculated as mean of SD from the five imputed datasets

**Table 3:** Ever-drinker and hazardous drinker prevalence estimates, and mean AUDIT-C score and weekly alcohol consumption among adults in Germany, stratified by region and federal states.

		Prevalence of ever-drinkers, % (95% CI)	Prevalence of hazardous drinkers, % (95% CI)	AUDIT-C score, mean (SD)	Weekly alcohol consumption in grams, mean (SD)
Germany (n = 11,127)		84.7 (84.1, 85.4)	19.4 (18.6, 20.1)	2.8 (2.16)	37.9 (56.43)
South West (n = 4,670)		83.2 (82.1, 84.2)	13.1 (12.1, 14.1)	2.4 (1.88)	29.6 (42.57)
	Baden-Wuerttemberg (n = 1,383)	82.9 (80.9, 84.9)	12.0 (10.3, 13.8)	2.4 (1.89)	29.4 (44.91)
	Bavaria (n = 1,751)	85.2 (83.5, 86.8)	11.8 (10.3, 13.3)	2.3 (1.74)	27.9 (38.61)
	Hesse (n = 807)	77.3 (74.4, 80.2)	13.8 (11.4, 16.2)	2.3 (2.06)	29.2 (44.04)
	Rhineland-Palatinate (n = 583)	85.0 (82.1, 87.9)	20.3 (17.1, 23.6)	2.8 (2.01)	35.8 (47.57)
	Saarland (n = 146)	87.3 (81.8, 92.7)	5.9 (2.1, 9.8)	2.5 (1.63)	28.5 (32.86)
North East (n = 931)		87.5 (85.3, 89.6)	23.8 (21.0, 26.5)	3.0 (2.29)	44.6 (71.28)
	Brandenburg (n = 356)	87.0 (83.5, 90.5)	30.4 (25.6, 35.2)	3.4 (2.66)	60.4 (96.54)
	Mecklenburg-Western Pomerania (n = 243)	92.1 (88.7, 95.5)	20.9 (15.8, 26.1)	2.9 (1.91)	36.6 (47.64)
	Saxony-Anhalt (n = 333)	84.6 (80.7, 88.5)	18.8 (14.5, 23.0)	2.6 (2.04)	33.6 (46.81)
North West (n = 4,575)		85.2 (84.2, 86.3)	23.8 (22.6, 25.0)	3.1 (2.25)	42.2 (57.86)
	Berlin (n = 455)	78.6 (74.9, 82.4)	30.4 (26.2, 34.7)	3.2 (2.51)	47.2 (60.29)
	Bremen (n = 89)	90.5 (84.4, 96.7)	31.6 (21.7, 41.4)	3.5 (2.10)	55.1 (66.79)
	Hamburg (n = 246)	88.6 (84.6, 92.6)	12.8 (8.6, 17.0)	2.8 (1.78)	30.1 (30.26)
	Lower Saxony (n = 1,030)	84.0 (81.8, 86.2)	17.3 (15.0, 19.6)	2.9 (2.11)	35.9 (51.59)
	North Rhine-Westphalia (n = 2,330)	85.7 (84.3, 87.2)	26.6 (24.8, 28.4)	3.2 (2.31)	45.4 (63.78)
	Schleswig-Holstein (n = 424)	89.6 (86.6, 92.5)	21.8 (17.9, 25.8)	3.1 (2.14)	38.6 (40.69)
South East (n = 951)		87.3 (85.2, 89.4)	24.5 (21.8, 27.2)	3.2 (2.54)	51.7 (81.43)
	Saxony (n = 615)	86.2 (83.4, 88.9)	26.3 (22.9, 29.8)	3.3 (2.66)	55.0 (84.87)
	Thuringia (n = 336)	89.5 (86.2, 92.8)	21.1 (16.7, 25.5)	3.0 (2.30)	45.8 (74.48)

Data weighted to be representative of the German population.

Complete cases for all variables of interest except for depression and anxiety where multiple imputation was used.

### 3.3 Average alcohol consumption (AUDIT-C) score and weekly alcohol consumption

The mean AUDIT-C score among adults in Germany was 2.8 (SD = 2.16; median = 2.00, IQR = 1.00–4.00) and the mean weekly alcohol consumption was 37.9g of alcohol (SD = 56.43; median = 18.54, IQR = 7.88–46.28). The mean AUDIT-C scores and weekly alcohol consumption data are presented stratified by the predictor variables in Table 2, and by federal state and region in Table 3. The sensitivity analysis with complete cases of all variables (including depression and anxiety) showed a similar pattern of results (Supplementary Table 1 and Supplementary Table 2).

### 3.4 Factors associated with alcohol consumption (AUDIT-C) score

Alcohol consumption, in terms of AUDIT-C score, was independently positively associated with having medium and high educational qualifications (compared with low), monthly income, being a current smoker, having anxiety, and living in North East, North West and South East Germany (compared with South West), and independently negatively associated with age, being female, having depression and survey wave (October to January and February to May, compared with June to September) (Table 4). The sensitivity analyses with i) Berlin coded as North East Germany showed the same pattern of results (Supplementary Table 3) and ii) including complete cases of all variables (including depression and anxiety) showed a similar pattern of results (Supplementary Table 4).

**Table 4:** Factors associated with level of alcohol consumption in terms of AUDIT-C score and prevalence of hazardous drinking among adults in Germany.

		AUDIT-C score				Prevalence of hazardous drinking			
		Unadjusted B (95% CI)	p	Adjusted B (95% CI)	p	Unadjusted OR (95% CI)	p	Adjusted OR (95% CI)	p
Age^a^		−0.30 (-0.34, −0.26)	<0.001	−0.17 (-0.21, −0.13)	<0.001	0.75 (0.72, 0.79)	<0.001	0.84 (0.79, 0.89)	<0.001
*Sex*									
	Male*								
	Female	−1.33 (-1.41, −1.25)	<0.001	−1.21 (-1.28, −1.14)	<0.001	0.26 (0.23, 0.29)	<0.001	0.26 (0.24, 0.29)	<0.001
*Marital status*									
	Not married*								
	Married	−0.08 (-0.16, 0.00)	0.051	0.001 (-0.08, 0.08)	0.983	0.78 (0.71, 0.86)	<0.001	0.92 (0.83, 1.03)	0.154
*Educational qualifications*									
	Low*								
	Medium	0.16 (0.08, 0.24)	<0.001	0.12 (0.02, 0.21)	0.014	1.07 (0.97, 1.18)	0.168	0.95 (0.83, 1.08)	0.408
	High	0.29 (0.20, 0.38)	<0.001	0.11 (0.01, 0.21)	0.034	1.11 (1.01, 1.23)	0.038	0.94 (0.82, 1.08)	0.395
Monthly income (per €1000)^b^		0.35 (0.30, 0.40)	<0.001	0.31 (0.26, 0.36)	<.001	1.20 (1.13, 1.27)	<.001	1.26 (1.18, 1.35)	<.001
*Smoking status*									
	Never and ex-smokers*								
	Current smoker	1.13 (1.05, 1.22)	<0.001	0.94 (0.86, 1.02)	<0.001	3.27 (2.97, 3.61)	<0.001	2.90 (2.61, 3.23)	<0.001
*Federal state*									
	North Rhine-Westphalia*								
	Baden-Wuerttemberg	−0.34 (-0.46, −0.22)	<0.001	–	–	0.66 (0.56, 0.77)	<0.001	–	–
	Bavaria	−0.50 (-0.61, −0.38)	<0.001	–	–	0.56 (0.48, 0.65)	<0.001	–	–
	Hesse	−0.67 (-0.82, −0.53)	<0.001	–	–	0.51 (0.41, 0.63)	<0.001	–	–
	Rhineland-Palatinate	0.14 (-0.05, 0.32)	0.143	–	–	1.23 (1.00, 1.50)	0.047	–	–
	Saarland	−0.29 (-0.56, −0.02)	0.034	–	–	0.38 (0.24, 0.59)	<0.001	–	–
	Brandenburg	0.47 (0.20, 0.73)	0.001	–	–	1.61 (1.22, 2.13)	0.001	–	–
	Mecklenburg-Western Pomerania	−0.03 (-0.27, 0.22)	0.823	–	–	0.86 (0.64, 1.16)	0.325	–	–
	Saxony-Anhalt	−0.30 (-0.55, −0.04)	0.022	–	–	0.78 (0.56, 1.07)	0.126	–	–
	Berlin	0.52 (0.34, 0.69)	<0.001	–	–	1.97 (1.64, 2.35)	<0.001	–	–
	Bremen	1.09 (0.68, 1.49)	<0.001	–	–	2.49 (1.69, 3.69)	<0.001	–	–
	Hamburg	−0.09 (-0.34, 0.16)	0.479	–	–	0.50 (0.34, 0.72)	<0.001	–	–
	Lower Saxony	0.18 (0.04, 0.32)	0.012	–	–	0.99 (0.84, 1.17)	0.935	–	–
	Schleswig-Holstein	0.18 (-0.03, 0.39)	0.085	–	–	1.28 (1.02, 1.61)	0.034	–	–
	Saxony	0.56 (0.37, 0.76)	<0.001	–	–	1.53 (1.25, 1.88)	<0.001	–	–
	Thuringia	0.09 (-0.13, 0.31)	0.430	–	–	1.19 (0.93, 1.52)	0.178	–	–
*Region of Germany*									
	South West*	–	–			–	–		
	North East	–	–	0.43 (0.29, 0.58)	<0.001	–	–	1.61 (1.31, 1.97)	<0.001
	North West	–	–	0.47 (0.39, 0.55)	<0.001	–	–	1.79 (1.59, 2.00)	<0.001
	South East	–	–	0.79 (0.64, 0.93)	<0.001	–	–	2.31 (1.91, 2.80)	<0.001
*Depression*									
	No*								
	Yes^c^	−0.30 (-0.54, −0.06)	0.015	−0.22 (-0.43, −0.02)	0.034	1.03 (0.83, 1.28)	0.780	0.97 (0.74, 1.28)	0.834
*Anxiety*									
	No*								
	Yes^d^	0.001 (-0.25, 0.25)	0.993	0.26 (0.02, 0.50)	0.033	1.28 (0.98, 1.67)	0.072	1.38 (1.00, 1.91)	0.054
*Survey wave*									
	Jun to Sep*								
	Oct to Jan	−0.10 (-0.18, −0.01)	0.022	−0.18 (-0.27, −0.09)	<0.001	0.87 (0.79, 0.96)	0.008	0.81 (0.72, 0.92)	0.001
	Feb to May	−0.05 (-0.14, 0.03)	0.231	−0.16 (-0.25, −0.07)	0.001	1.0.1 (0.92, 1.12)	0.819	0.91 (0.81, 1.03)	0.135

Complete cases for all variables of interest except for depression and anxiety where multiple imputation was used

* Reference level

a Age variable transformed (divided by the standard deviation[=18.43])

b Range from 0 (€0 income) to 7 (€7,000 or more)

c A score of 3 or more on the depression subscale of the PHQ-4

d A score of 3 or more on the anxiety subscale of the PHQ-4.

Weekly alcohol consumption (in grams of alcohol) was independently positively associated with monthly income, being a current smoker, living in North East, North West and South East Germany (compared with South West) and having anxiety, and independently negatively associated with being female and survey wave (October to January and February to May, compared with June to September) (Supplementary Table 5).

### 3.5 Factors associated with prevalence of hazardous drinking

The prevalence of hazardous drinking was independently positively associated with monthly income, being a current smoker, and living in North East, North West and South East Germany (compared with South West), and independently negatively associated with age, being female, and survey wave (October to January compared with June to September) (Table 4). The sensitivity analyses with i) Berlin coded as North East Germany showed the same pattern of results (Supplementary Table 6) and ii) including complete cases of all variables (including depression and anxiety) showed a similar pattern of results (Supplementary Table 7).

## 4 Discussion

### 4.1 Summary of findings

Among a representative sample of adults in Germany between 2018 and 2019, the prevalence of ever-drinking was 85% and the prevalence of hazardous drinking was 19%. The mean AUDIT-C score was 2.8 indicating low-risk alcohol consumption, and the mean weekly consumption was 37.9g of alcohol which equates to about 3.4 German units (1 German unit = 10–12g of alcohol) per week. Both the prevalence of hazardous drinking and AUDIT-C score were lower among older adults and among females. The prevalence of hazardous drinking and alcohol consumption (AUDIT-C) score were positively associated with monthly income, being a current smoker, and living in North East, North West and South East Germany (compared with South West). AUDIT-C score was also independently positively associated with having medium and high, compared with low, educational qualifications, and anxiety. AUDIT-C score was also lower among those with depression.

The mean AUDIT-C score among our sample indicates low-risk alcohol consumption across the whole population, but this can obscure subgroups with much higher consumption. In this study, we defined prevalence of hazardous drinking based on the AUDIT-C score. The prevalence rates of hazardous drinking in our study are similar to the prevalence rates found in another survey from 2018, which used daily consumption of alcohol as the definition for hazardous drinking (Atzendorf et al., 2019). However, an earlier study from 2012, from the GEDA, that used the AUDIT-C score to define hazardous drinking, found higher prevalence rates of hazardous drinking (32% for men and 21% for women) (John, Hanke, & Freyer-Adam, 2018). Data from 2008 to 2011 from the DEGS showed a much higher prevalence of hazardous drinking (26% for women and 42% for men) when defined using AUDIT-C score compared with average alcohol consumed per day (12/24g for women/men; 13.1% for women and 18.5% for men) (Lange et al., 2016). This suggests that the differences in these prevalence rates might be related to how hazardous drinking is operationalised in a survey. This highlights the importance of ongoing and regular data collection using consistent methodology, both in terms of the measures used and study procedure, for understanding the trends in alcohol consumption and for evaluating the effectiveness of any changes in alcohol policy in Germany.

In the current study, older adults were less likely to be hazardous drinkers and the alcohol consumption score also decreased with age. Alcohol consumption tends to decrease with age due to physiological, metabolic and medication profile changes (Cederbaum, 2012, Immonen et al., 2013, Meier and Seitz, 2008, St-Onge and Gallagher, 2010) and because alcohol consumption is associated with increased mortality (Fullman et al., 2018, Sathyanarayana Rao and Andrade, 2016, Wood et al., 2018). Previous research found that the prevalence of risky drinking in Germany was highest among those aged 45–64 years (Lange et al., 2017), though Germany also displayed a moderate cross-sectional age-related decline in the prevalence of heavy drinking (Calvo et al., 2020). This highlights the importance of using standardised data collection methods to more accurately assess and better understand trends in alcohol consumption in Germany.

The present study found that men had a signficiantly higher prevalence of hazardous drinking and higher alcohol consumption scores than women, in line with previous findings (Atzendorf et al., 2020, Bott et al., 2005, Halme et al., 2008, Lange, Mankertz, & Kuntz, 2017, Lange et al., 2016). Higher SEP was positively associated with alcohol consumption (AUDIT-C) score and prevalence of hazardous drinking, which was also in line with previous findings from Germany (Atzendorf et al., 2020, Pabst et al., 2019) and is consistent with the alcohol harm paradox (Bellis et al., 2016). These findings are reassuring in that existing alcohol-related health inequalities are unlikely to have widened, as those of low SEP are more at risk of alcohol-related harm for the same level of alcohol consumption compared with those of high SEP (Bellis et al., 2016). However, it is important for future research to assess drinking patterns that also contribute to socioeconomic inequalities in alcohol-related mortality (Boyd et al., 2021, Probst et al., 2020).

Being a current smoker was associated with both the prevalence of hazardous drinking and alcohol consumption score, in line with previous findings (Beard et al., 2017, Falk et al., 2006, Hasin et al., 2007, Lange et al., 2016). Having anxiety was positively associated with the alcohol consumption score, comparable with previous findings (Bott et al., 2005). However, unlike previous research (Bott et al., 2005), this study found that alcohol consumption score was lower among those with depression. These differences may be due to methodological differences in the assessment of psychiatric diagnoses; assessment was based on the clinical DSM-IV criteria in the study of Bott et al. (Bott et al., 2005), while our study used a brief screening instrument, the PHQ-4 (Kroenke et al., 2009, Löwe et al., 2010) to assess symptoms of anxiety and depression in the past two weeks.

Region of Germany was also associated with the alcohol consumption score and prevalence of hazardous drinking; living in North East, North West and South East Germany, compared with the South West, was associated with higher prevalences of hazardous drinking and higher alcohol consumption scores. These findings may reflect differences in social patterns and culture not captured by measures of SEP. Our results are partly in line with data from the 2015 ESA which found prevalence of at-risk alcohol consumption was higher in East, compared with West, Germany though also that prevalence was higher in South, compared with North, Germany (Atzendorf et al., 2020). However, the sampling procedure of the study aims to produce a sample which is representative for the total population, but not necessarily for each individual federal state. Therefore, regional prevalence data should be interpreted with caution.

### 4.2 Strengths and limitations

A key strength of this study is the representative sample and the presence of a broad range of relevant characteristics that were collected recently among the general population of adults in Germany. Other studies that have reported alcohol consumption in Germany are not as recent or do not include standardised measures, such as the AUDIT-C, which allows for international comparisons.

However, we were unable to conduct a time-series trend analysis, which would require a longer time period than we have data available for and so cannot assess whether there have been any trends in prevalence rates. Another limitation is that there was some missing data for depression and anxiety. We used multiple imputation to impute the missing values for these variables though the data are unlikely to be missing at random or completely at random as people with depression and/or anxiety might be more likely to not respond to these questions than those without. Furthermore, household surveys asking questions about typical quantities of alcohol consumed can lead to under-estimates (Stockwell et al., 2004), and greater under-reporting of alcohol consumption may be associated with heavy drinking and non-routine drinking patterns (Boniface, Kneale, & Shelton, 2014). Therefore, the prevalence rates and level of alcohol consumption reported in this study could be under-estimates. Another limitation of the study is that the methodology of the market research institute conducting the survey does not allow for a calculation of the response rate and therefore we are unable to compare responders and non-responders.

A review of research findings on the AUDIT recommended using gender specific cut-offs with the AUDIT-C to identify hazardous drinking (Reinert & Allen, 2007). A potential limitation of this study is that it used the same AUDIT-C cut-off for hazardous drinking for both men and women (Rumpf et al., 2002, National Institute for Health and Care, 2018) as the proportion of women identified as hazardous drinkers may be higher than these findings suggest. However, these cut-offs were not used for the purposes of screening and providing brief advice to individuals, so the issue of a false negative is less critical. Furthermore, there is evidence that the AUDIT is unidimensional with measurement invariance across gender, meaning that direct comparisons between men and women can be made (Skogen, Thørrisen, Olsen, Hesse, & Aas, 2019), and some countries, such as the UK, have set universal low-risk drinking guidance as they judged the risks for men and women to be similar (Department of Health, 2016).

Whilst the AUDIT-C is a useful measure of alcohol consumption (Bradley et al., 2007, Bush et al., 1998, Reinert and Allen, 2007), it is also important to evaluate drinking patterns in more detail and heavy episodic drinking to more accurately estimate alcohol-related risk and mortality (Bobak et al., 2004, Rehm et al., 2003, Rehm, Greenfield, & Rogers, 2001). Future research should look to assess drinking patterns and heavy episodic drinking to better estimate alcohol-related problems at the population level.

### 4.3 Conclusions

Among a representative sample of adults in Germany between 2018 and 2019, the prevalence of ever-drinking was 85%, around one fifth were hazardous drinkers, and the mean AUDIT-C score was 2.8, indicating generally low-risk alcohol consumption. Prevalence of hazardous drinking and alcohol consumption scores were associated with being younger, male, a current smoker, of high SEP, and living in North East, North West and South East Germany (compared with South West). This highlights that there are certain groups in Germany who may benefit from targeted alcohol reduction policies and support.

## 5 Author’s Contribution

All authors conceptualised the study, and contributed to and approved the final manuscript.

CG conducted the statistical analysis and wrote the first draft of the manuscript. DK acquired funding for the DEBRA study.

## Funding

The DEBRA study was funded from 2016 to 2019 (waves 1–18) by the Ministry of Innovation, Science and Research of the German State of North Rhine–Westphalia (MIWF) in the context of the “NRW Rückkehrprogramm” (the North Rhine–Westphalian postdoc return program). Since 2019 (wave 19 onwards), the study has been funded by the German Federal Ministry of Health. CG receives salary support from Cancer Research UK (C1417/A22962) and NIHR (NIHR127651). The funders had no role in the study design, collection, analysis or interpretation of the data, writing the manuscript, or the decision to submit the paper for publication.

### CRediT authorship contribution statement

**Claire Garnett:** Conceptualization, Formal analysis, Writing – original draft, Writing – review & editing. **Sabrina Kastaun:** Conceptualization, Writing – review & editing. **Jamie Brown:** Conceptualization, Writing – review & editing. **Daniel Kotz:** Conceptualization, Writing – review & editing, Funding acquisition.

## Declaration of Competing Interest

The authors declare the following financial interests/personal relationships which may be considered as potential competing interests: CG, SK and DK have no conflicts of interest to declare. JB has received unrestricted funding related to smoking cessation research.
