# Supplementary material for: Alcohol consumption and associations with sociodemographic and health-related characteristics in Germany: A population survey
Source: Addict Behav. 2022 Feb;125:107159. doi: 10.1016/j.addbeh.2021.107159 (PMC8642732; doi:10.1016/j.addbeh.2021.107159)
Supplement: Supplementary Data 1 [file mmc1.docx]

# Supplementary File 1

## Sampling method

All communities within Germany are stratified by federal state, administrative district, and type of community. The geographical area of Germany was divided into 53,000 small areas to determine the primary sampling units. For each of these small areas, the sample size was determined in proportion to the total number of households in the area relative to the total number of households in the whole population. ﻿The secondary sampling units are represented through private households and are selected by a random walk procedure. The tertiary sampling units are the target persons themselves and are selected using a random process that gives an equal chance of selection to every eligible person within a household.

## The AUDIT-C measure

**English version:**

1. How often do you have a drink containing alcohol, for example, a glass of wine, beer, mixed drinks, schnapps or liqueur?
   Answer: Never (score 0), Monthly or less (score 1), 2–4 times per month (score 2), 2–3 times per week (score 3), 4+ times per week (score 4).
2. How many units of alcohol do you drink on a typical day when you are drinking? By an alcoholic drink we again mean a small bottle of beer of 0.33 litres, a small glass of wine of 0.125 litres, a glass of sparkling wine, a double schnapps or a bottle of alcopops.
   Answer: 1–2 (score 0), 3–4 (score 1), 5–6 (score 2), 7–9 (score 3), 10+ (score 4).
3. How often do you have 6 or more drinks on a single occasion? By an alcoholic drink we again mean a small bottle of beer of 0.33 litres, a small glass of wine of 0.125 litres, a glass of sparkling wine, a double schnapps or a bottle of alcopops.
   Answer: Never (score 0), Less than monthly (score 1), Monthly (score 2), Weekly (score 3), Daily or almost daily (score 4).

**German version:**

In unserem heutigen Leben gibt es zahlreiche Gelegenheiten, in denen ein Glas Bier, Wein oder Schnaps fast dazu gehört (z.B. beim Abendessen oder auf einer Party). Im Folgenden möchten wir Ihnen daher Fragen zu Ihrem Konsum alkoholischer Getränke stellen.

1. Wie oft nehmen Sie ein alkoholisches Getränk also zum Beispiel ein Glas Wein, Bier, Mixgetränke, Schnaps oder Likör zu sich?
   Antwort: nie (Wert 0), 1 mal pro Monat oder seltener (1), 2-4 mal im Monat (2), 2-3 mal die Woche (3), 4 mal die Woche oder öfter (4).
2. Wenn Sie Alkohol trinken, wie viele alkoholische Getränke trinken Sie dann üblicherweise an einem Tag? Mit einem alkoholischen Getränk meinen wir eine kleine Flasche Bier mit 0,33 Liter, ein kleines Glas Wein mit 0,125 Liter, ein Glas Sekt, einen doppelten Schnaps oder eine Flasche Alkopops.
   Antwort: 1 bis 2 (Wert 0), 3 bis 4 (1), 5 bis 6 (2), 7 bis 9 (3), 10 oder mehr alkoholische Getränke (4).
3. Wie oft trinken Sie 6 oder mehr Gläser Alkohol bei einer Gelegenheit (z.B. beim Abendessen oder auf einer Party)? Mit einem alkoholischen Getränk meinen wir wieder eine kleine Flasche Bier mit 0,33 Liter, ein kleines Glas Wein mit 0,125 Liter, ein Glas Sekt, einen doppelten Schnaps oder eine Flasche Alkopops.
   Antwort: niemals (Wert 0), seltener als 1 mal im Monat (1), jeden Monat (2), jede Woche (3), jeden Tag oder fast jeden Tag (4).

## Weekly alcohol consumption measure

Level of alcohol consumption was transformed into an estimate of weekly alcohol consumption (in grams of alcohol per week) to provide a more interpretable measure. It was estimated from the AUDIT-C, as the product of frequency (item 1) by quantity (item 2) with adjustment for occasional heavy drinking (item 3) [1]. Each response option for each item of the AUDIT-C is given a value and then the weekly alcohol consumption equals *(item 1 value*item 2 value) + item 3 value* [2]. For example, the response option ‘2 to 3 times a week’ for item 1 is a value of 1.61, ‘1 to 2’ for item 2 is a value for 19.4, and ‘Less than monthly’ for item 3 is a value of 15.0, which results in a estimated weekly alcohol consumption of 46.3g of alcohol.

## Monthly net household income measure

As the needs and expenses of a household depend on the age and number of people living in it, we used an equalisation technique of the Organisation for Economic Co-operation and Development (OECD) (OECD-modified equivalence scale) to adjust income for household size and composition. Each member of a household received a different weighting (1.0 to the first adult [defined here as >=14 years] of a household, 0.5 to each further adult, and 0.3 to each child <14 years), and the net total household income was divided by the sum of the weightings to calculate a representative household income (details on the calculation are published here: <https://osf.io/e2nqr/>). We then used monthly net household income as a continuous variable coded from 0 (€0 income/month) to 7 (€7,000 or more/month).

## Depression and anxiety measures

Depression and anxiety were measured using the validated Patient Health Questionnaire-4 (PHQ*-*4) [3,4]. Respondents were asked: “How often have you felt affected by the following complaints over the last 2 weeks?” asked about i) “little interest or pleasure in doing things”, ii) “depression, melancholy or hopelessness”, iii) “nervousness, anxiety or on edge”, iv) “not being able to stop or control worrying”. There were four response options: a) not at all (coded 0); b) on several days (coded 1); c) on more than half of the days (coded 2); d) nearly every day (coded 3). Scores of 3 or above across the depression (i and ii) and anxiety subscales (iii and iv) indicated probable cases of depression and anxiety, respectively [3,4].

## Prevalence of ever-drinking and hazardous drinking, and alcohol consumption (AUDIT-C) score and weekly alcohol consumption among adolescents (aged 14-17)

A total of 272 adolescents (weighted n=352) aged from 14 to 17 years (inclusive) in the general population in Germany participated in the study between June/July 2018 and April/May 2019. The mean age was 15.6 years (SD=1.06), about half were female (52.5%, n=185), and 9.7% were current smokers (n=34). The prevalence of ever-drinking was 45.4% (n=160) and 6.8% (n=24) for hazardous drinking among adolescents in Germany. The mean AUDIT-C score was 1.3 (SD=1.92) and the mean weekly alcohol consumption was 13.8g (SD=29.64). The prevalence of ever- and hazardous drinking and alcohol consumption (AUDIT-C) score by region of Germany is reported in Supplementary Table 8.

## References

1. Dutey-Magni P, Brown J, Holmes J, Sinclair J. Concurrent validity of an Estimator of Weekly Alcohol Consumption (EWAC) based on the Extended AUDIT. Under Rev.

2. Dutey-Magni P. Estimator of weekly alcohol consumption [Internet]. GitHub. 2020. Available from: https://github.com/peterdutey/ewac-web

3. Kroenke K, Spitzer RL, Williams JBW, Löwe B. An ultra-brief screening scale for anxiety and depression: the PHQ-4. Psychosomatics. 2009;50(6):613–21.

4. Löwe B, Wahl I, Rose M, Spitzer C, Glaesmer H, Wingenfeld K, et al. A 4-item measure of depression and anxiety: validation and standardization of the Patient Health Questionnaire-4 (PHQ-4) in the general population. J Affect Disord. 2010 Apr;122(1–2):86–95.
