# Supplementary material for: Alcohol consumption and associations with sociodemographic and health-related characteristics in Germany: A population survey
Source: Addict Behav. 2022 Feb;125:107159. doi: 10.1016/j.addbeh.2021.107159 (PMC8642732; doi:10.1016/j.addbeh.2021.107159)
Supplement: Supplementary Data 2 [file mmc2.docx]

# Supplementary Tables

Supplementary Table 1: Ever-drinker and hazardous drinker prevalence estimates and mean AUDIT-C stratified by predictor variables among adults in Germany (complete cases for all predictor variables including depression and anxiety)

|  | | Prevalence of ever-drinkers, % (95% CI) | Prevalence of hazardous drinkers, % (95% CI) | AUDIT-C score, mean (SD) |
| --- | --- | --- | --- | --- |
| All respondents (n=9,937) | | 85.7 (85.0, 86.4) | 19.5 (18.8, 20.3) | 2.8 (2.13) |
| Age | |  |  |  |
|  | 18-24 (n=949) | 84.2 (81.9, 86.6) | 23.2 (20.5, 25.9) | 3.0 (2.30) |
|  | 25-34 (n=1,249) | 88.5 (86.7, 90.2) | 21.6 (19.3, 23.9) | 2.9 (2.19) |
|  | 35-44 (n=1,441) | 86.6 (84.9, 88.4) | 22.6 (20.4, 24.7) | 2.9 (2.17) |
|  | 45-54 (n=1,810) | 89.6 (88.2, 91.1) | 21.2 (19.3, 23.1) | 3.0 (2.02) |
|  | 55-64 (n=1,757) | 88.4 (86.9, 89.9) | 21.9 (20.0, 23.8) | 3.0 (2.17) |
|  | 65+ (n=2,730) | 80.1 (78.6, 81.6) | 13.2 (11.9, 14.4) | 2.4 (1.98) |
| Sex | |  |  |  |
|  | Male (n=4,745) | 90.4 (89.5, 91.2) | 30.0 (28.7, 31.3) | 3.5 (2.27) |
|  | Female (n=5,192) | 81.4 (80.4, 82.5) | 10.0 (9.2, 10.8) | 2.2 (1.75) |
| Marital status | |  |  |  |
|  | Not (n=4,312) | 83.8 (82.7, 84.9) | 21.1 (19.9, 22.4) | 2.8 (2.26) |
|  | Married (n=5,625) | 87.2 (86.3, 88.0) | 18.3 (17.3, 19.3) | 2.8 (2.02) |
| Educational qualifications | |  |  |  |
|  | Low (n=3,062) | 80.8 (79.4, 82.2) | 17.6 (16.3, 19.0) | 2.6 (2.22) |
|  | Medium (n=3,808) | 87.9 (86.9, 89.0) | 22.0 (20.7, 23.3) | 3.0 (2.18) |
|  | High (n=3,067) | 87.8 (86.6, 88.9) | 18.4 (17.1, 19.8) | 2.9 (1.94) |
| Monthly income (per €1000)^a^ | |  |  |  |
|  | Low (n=5,983) | 82.8 (81.9, 83.8) | 17.4 (16.4, 18.4) | 2.6 (2.11) |
|  | High (n=3,954) | 90.0 (89.1, 91.0) | 22.8 (21.5, 24.1) | 3.2 (2.11) |
| Smoking status | |  |  |  |
|  | Never and ex-smokers (n=6,924) | 84.5 (83.7, 85.4) | 13.9 (13.1, 14.7) | 2.5 (1.90) |
|  | Current smoker (n=3,013) | 88.4 (87.2, 89.5) | 32.6 (30.9, 34.3) | 3.5 (2.44) |
| Depression |  |  |  |  |
|  | No (n=9,491) | 86.4 (85.7, 87.1) | 19.6 (18.8, 20.4) | 2.8 (2.11) |
|  | Yes^b^ (n=446) | 70.8 (66.6, 75.0) | 18.3 (14.7, 21.9) | 2.5 (2.42) |
| Anxiety |  |  |  |  |
|  | No (n=9,640) | 86.1 (85.4, 86.8) | 19.5 (18.7, 20.3) | 2.8 (2.11) |
|  | Yes^c^ (n=297) | 72.5 (67.4, 77.6) | 20.3 (15.7, 24.9) | 2.7 (2.75) |

Data weighted to be representative of the German population

Complete cases for all variables of interest including depression and anxiety
^a^ Dichotomised based on the mean score of 1.69 (range from 0 [€0 income] to 7 [€7,000 or more])
^b^ A score of 3 or more on the depression subscale of the PHQ-4
^c^ A score of 3 or more on the anxiety subscale of the PHQ-4

Supplementary Table 2: Ever-drinker and hazardous drinker prevalence estimates and mean AUDIT-C score among adults in Germany, stratified by region and federal states (complete cases for all predictor variables including depression and anxiety)

|  | | Prevalence of ever-drinkers, % (95% CI) | Prevalence of hazardous drinkers, % (95% CI) | AUDIT-C score, mean (SD) |
| --- | --- | --- | --- | --- |
| All respondents (n=9,937) | | 85.7 (85.0, 86.4) | 19.5 (18.8, 20.3) | 2.8 (2.13) |
| South West (n=4,132) | | 84.3 (83.2, 85.4) | 13.1 (12.1, 14.2) | 2.4 (1.86) |
|  | Baden-Wuerttemberg (n=1,217) | 84.0 (82.0, 86.1) | 11.5 (9.7, 13.3) | 2.4 (1.89) |
|  | Bavaria (n=1,593) | 85.7 (83.9, 87.4) | 12.5 (10.8, 14.1) | 2.3 (1.74) |
|  | Hesse (n=728) | 81.7 (78.9, 84.5) | 14.6 (12.1, 17.2) | 2.4 (2.01) |
|  | Rhineland-Palatinate (n=455) | 83.7 (80.3, 87.1) | 19.7 (16.0, 23.4) | 2.7 (1.98) |
|  | Saarland (n=139) | 87.5 (82.0, 93.1) | 5.8 (1.9, 9.7) | 2.5 (1.62) |
| North East (n=838) | | 89.5 (87.4, 91.6) | 23.3 (20.4, 26.2) | 3.0 (2.13) |
|  | Brandenburg (n=309) | 87.6 (83.9, 91.3) | 28.5 (23.4, 33.6) | 3.2 (2.42) |
|  | Mecklenburg-Western Pomerania (n=232) | 92.8 (89.4, 96.1) | 21.8 (16.4, 27.1) | 2.9 (1.91) |
|  | Saxony-Anhalt (n=297) | 88.9 (85.3, 92.5) | 19.0 (14.6, 23.5) | 2.7 (1.95) |
| North West (n=4,074) | | 85.9 (84.9, 87.0) | 24.2 (22.9, 25.6) | 3.1 (2.23) |
|  | Berlin (n=426) | 78.6 (74.7, 82.5) | 31.8 (27.3, 36.2) | 3.3 (2.54) |
|  | Bremen (n=78) | 91.6 (85.4, 97.9) | 32.9 (22.3, 43.6) | 3.6 (2.04) |
|  | Hamburg (n=220) | 88.4 (84.2, 92.7) | 13.2 (8.7, 17.8) | 2.8 (1.79) |
|  | Lower Saxony (n=864) | 84.9 (82.5, 87.3) | 16.8 (14.3, 19.3) | 2.9 (2.01) |
|  | North Rhine-Westphalia (n=2,087) | 86.6 (85.1, 88.0) | 26.9 (25.0, 28.8) | 3.2 (2.30) |
|  | Schleswig-Holstein (n=398) | 90.3 (87.4, 93.2) | 22.7 (18.5, 26.8) | 3.1 (2.16) |
| South East (n=893) | | 87.3 (85.1, 89.5) | 24.2 (21.4, 27.1) | 3.1 (2.47) |
|  | Saxony (n=563) | 86.0 (83.1, 88.8) | 25.9 (22.2, 29.5) | 3.2 (2.55) |
|  | Thuringia (n=330) | 89.6 (86.3, 92.9) | 21.4 (17.0, 25.9) | 3.0 (2.32) |

Data weighted to be representative of the German population
Complete cases for all variables of interest including depression and anxiety

Supplementary Table 3: Factors associated with level of alcohol consumption in terms of AUDIT-C score among adults in Germany with Berlin coded as North East Germany

|  | | | Adjusted | |
| --- | --- | --- | --- | --- |
|  | | | B (95% CI) | p |
| Age^a^ | | | -0.17 (-0.21, -0.14) | <.001 |
| Sex | | |  |  |
|  | Male* | |  |  |
|  | Female | | -1.21 (-1.28, -1.14) | <.001 |
| Marital status | | |  |  |
|  | Not married* | |  |  |
|  | Married | | 0.002 (-0.07, 0.08) | .951 |
| Educational qualifications | | |  |  |
|  | Low* | |  |  |
|  | Medium | | 0.11 (0.02, 0.20) | .021 |
|  | High | | 0.11 (0.004, 0.21) | .041 |
| Monthly income (per €1000)^b^ | | | 0.32 (0.27, 0.37) | <.001 |
| Smoking status | | |  |  |
|  | Never and ex-smokers* | |  |  |
|  | Current smoker | | 0.94 (0.86, 1.02) | <.001 |
| Region of Germany | | |  |  |
|  | South West* | |  |  |
|  | North East | | 0.50 (0.39, 0.62) | <.001 |
|  | North West | | 0.45 (0.37, 0.54) | <.001 |
|  | South East | | 0.79 (0.65, 0.94) | <.001 |
| Depression | | |  |  |
|  | No* | |  |  |
|  | Yes^c^ | | -0.23 (-0.43, -0.02) | .030 |
| Anxiety | | |  |  |
|  | No* | |  |  |
|  | Yes^d^ | | 0.26 (0.02, 0.50) | .033 |
| Survey wave | | |  |  |
|  | | Jun to Sep* |  |  |
|  | | Oct to Jan | -0.18 (-0.27, -0.09) | <.001 |
|  | | Feb to May | -0.16 (-0.25, -0.07) | .001 |

Complete cases for all variables of interest except for depression and anxiety where multiple imputation was used
* Reference level
^a^ Age variable transformed (divided by the standard deviation[=18.43])
^b^ Range from 0 (€0 income) to 7 (€7,000 or more)

^c^ A score of 3 or more on the depression subscale of the PHQ-4
^d^ A score of 3 or more on the anxiety subscale of the PHQ-4

Supplementary Table 4: Factors associated with level of alcohol consumption in terms of AUDIT-C score among adults in Germany (complete cases for all predictor variables including depression and anxiety)

|  | | | Unadjusted | | Adjusted | |
| --- | --- | --- | --- | --- | --- | --- |
|  | | | B (95% CI) | p | B (95% CI) | p |
| Age^a^ | | | -0.27 (-0.31, -0.23) | <.001 | -0.15 (-0.19, -0.11) | <.001 |
| Sex | | |  |  |  |  |
|  | Male* | |  |  |  |  |
|  | Female | | -1.33 (-1.40, -1.25) | <.001 | -1.21 (-1.29, -1.14) | <.001 |
| Marital status | | |  |  |  |  |
|  | Not married* | |  |  |  |  |
|  | Married | | -0.04 (-0.13, 0.04) | .311 | 0.01 (-0.07, 0.09) | .806 |
| Educational qualifications | | |  |  |  |  |
|  | Low* | |  |  |  |  |
|  | Medium | | 0.21 (0.12, 0.29) | <.001 | 0.18 (0.08, 0.27) | <.001 |
|  | High | | 0.25 (0.16, 0.34) | <.001 | 0.11 (0.01, 0.22) | .038 |
| Monthly income (per €1000)^b^ | | | 0.36 (0.31, 0.41) | <.001 | 0.32 (0.27, 0.37) | <.001 |
| Smoking status | | |  |  |  |  |
|  | Never or ex-smoker* | |  |  |  |  |
|  | Current smoker | | 1.09 (1.01, 1.18) | <.001 | 0.90 (0.82, 0.99) | <.001 |
| Federal state | | |  |  |  |  |
|  | North Rhine-Westphalia* | |  |  |  |  |
|  | Baden-Wuerttemberg | | -0.36 (-0.49, -0.24) | <.001 | - | - |
|  | Bavaria | | -0.46 (-0.57, -0.34) | <.001 | - | - |
|  | Hesse | | -0.59 (-0.74, -0.44) | <.001 | - | - |
|  | Rhineland-Palatinate | | 0.09 (-0.11, 0.29) | .368 | - | - |
|  | Saarland | | -0.32 (-0.60, -0.05) | .020 | - | - |
|  | Brandenburg | | 0.32 (0.04, 0.60) | .028 | - | - |
|  | Mecklenburg-Western Pomerania | | -0.01 (-0.25, 0.24) | .964 | - | - |
|  | Saxony-Anhalt | | -0.26 (-0.52, -0.01) | .045 | - | - |
|  | Berlin | | 0.59 (0.42, 0.77) | <.001 | - | - |
|  | Bremen | | 0.95 (0.51, 1.39) | <.001 | - | - |
|  | Hamburg | | -0.04 (-0.30, 0.22) | .779 | - | - |
|  | Lower Saxony | | 0.10 (-0.05, 0.26) | .183 | - | - |
|  | Schleswig-Holstein | | 0.27 (0.05, 0.48) | .014 | - | - |
|  | Saxony | | 0.26 (0.06, 0.47) | .011 | - | - |
|  | Thuringia | | 0.11 (-0.11, 0.33) | .312 | - | - |
| Region of Germany | | |  |  |  |  |
|  | South West* | | - | - |  |  |
|  | North East | | - | - | 0.43 (0.29, 0.58) | <.001 |
|  | North West | | - | - | 0.52 (0.43, 0.60) | <.001 |
|  | South East | | - | - | 0.66 (0.51, 0.80) | <.001 |
| Depression | | |  |  |  |  |
|  | No* | |  |  |  |  |
|  | Yes^c^ | | -0.30 (-0.48, -0.12) | .001 | -0.23 (-0.42, -0.04) | .020 |
| Anxiety | | |  |  |  |  |
|  | No* | |  |  |  |  |
|  | Yes^d^ | | -0.13 (-0.35, 0.09) | .240 | 0.14 (-0.09, 0.38) | .228 |
| Survey wave | | |  |  |  |  |
|  | | Jun to Sep* |  |  |  |  |
|  | | Oct to Jan | -0.07 (-0.16, 0.02) | .115 | -0.16 (-0.25, -0.07) | .001 |
|  | | Feb to May | -0.06 (-0.15, 0.03) | .205 | -0.16 (-0.25, -0.07) | .001 |

Complete cases for all variables of interest including depression and anxiety
* Reference level
^a^ Age variable transformed (divided by the standard deviation[=18.43])
^b^ Range from 0 (€0 income) to 7 (€7,000 or more)
^c^ A score of 3 or more on the depression subscale of the PHQ-4
^d^ A score of 3 or more on the anxiety subscale of the PHQ-4

Supplementary Table 5: Factors associated with level of alcohol consumption in terms of weekly alcohol consumption (grams of alcohol) among adults in Germany

|  | | | Unadjusted | | Adjusted | |
| --- | --- | --- | --- | --- | --- | --- |
|  | | | B (95% CI) | p | B (95% CI) | p |
| Age^a^ | | | -3.44 (-4.45, -2.43) | <.001 | -0.84 (-1.89, 0.21) | .118 |
| Sex | | |  |  |  |  |
|  | Male* | |  |  |  |  |
|  | Female | | -27.28 (-29.27, -25.29) | <.001 | -25.42 (-27.38, -23.46) | <.001 |
| Marital status | | |  |  |  |  |
|  | Not married* | |  |  |  |  |
|  | Married | | -2.73 (-4.77, -0.68) | .009 | -0.81 (-2.84, 1.22) | .432 |
| Educational qualifications | | |  |  |  |  |
|  | Low* | |  |  |  |  |
|  | Medium | | 1.00 (-1.14, 3.13) | .359 | 0.23 (-2.24, 2.71) | .854 |
|  | High | | 3.33 (1.10, 5.56) | .003 | 1.41 (-1.32, 4.13) | .312 |
| Monthly income (per €1000)^b^ | | | 4.03 (2.77, 5.28) | <.001 | 4.16 (2.83, 5.49) | <.001 |
| Smoking status | | |  |  |  |  |
|  | Never or ex-smoker* | |  |  |  |  |
|  | Current smoker | | 24.10 (21.93, 26.28) | <.001 | 21.29 (19.09, 23.50) | <.001 |
| Federal state | | |  |  |  |  |
|  | North Rhine-Westphalia* | |  |  |  |  |
|  | Baden-Wuerttemberg | | -5.94 (-9.00, -2.87) | <.001 | - | - |
|  | Bavaria | | -8.17 (-11.02, -5.32) | <.001 | - | - |
|  | Hesse | | -11.20 (-14.96, -7.45) | <.001 | - | - |
|  | Rhineland-Palatinate | | 0.004 (-4.63, 4.64) | .999 | - | - |
|  | Saarland | | -7.03 (-13.89, -0.16) | .045 | - | - |
|  | Brandenburg | | 17.34 (10.51, 24.17) | <.001 | - | - |
|  | Mecklenburg-Western Pomerania | | -1.86 (-8.12, 4.40) | .561 | - | - |
|  | Saxony-Anhalt | | -5.48 (-11.93, 0.98) | .096 | - | - |
|  | Berlin | | 11.66 (7.15, 16.18) | <.001 | - | - |
|  | Bremen | | 25.16 (14.72, 35.59) | <.001 | - | - |
|  | Hamburg | | -7.23 (-13.56, -0.91) | .025 | - | - |
|  | Lower Saxony | | 1.22 (-2.42, 4.86) | .511 | - | - |
|  | Schleswig-Holstein | | -1.55 (-6.88, 3.77) | .568 | - | - |
|  | Saxony | | 20.66 (15.71, 25.62) | <.001 | - | - |
|  | Thuringia | | 2.73 (-2.94, 8.40) | .346 | - | - |
| Region of Germany | | |  |  |  |  |
|  | South West* | | - | - |  |  |
|  | North East | | - | - | 8.54 (4.66, 12.43) | <.001 |
|  | North West | | - | - | 6.61 (4.48, 8.74) | <.001 |
|  | South East | | - | - | 19.35 (15.51, 23.19) | <.001 |
| Depression | | |  |  |  |  |
|  | No* | |  |  |  |  |
|  | Yes^c^ | | 4.94 (-0.44, 10.33) | .071 | -1.69 (-7.43, 4.05) | .558 |
| Anxiety | | |  |  |  |  |
|  | No* | |  |  |  |  |
|  | Yes^d^ | | 15.53 (9.18, 21.88) | <.001 | 16.93 (10.84, 23.01) | <.001 |
| Survey wave | | |  |  |  |  |
|  | | Jun to Sep* |  |  |  |  |
|  | | Oct to Jan | -3.52 (-5.68, -1.35) | .001 | -5.30 (-7.67, -2.94) | <.001 |
|  | | Feb to May | -1.13 (-3.31, 1.05) | .309 | -3.96 (-6.34, 1.58) | .001 |

Complete cases for all variables of interest except depression and anxiety where multiple imputation was used
* Reference level
^a^ Age variable transformed (divided by the standard deviation[=18.43])
^b^ Range from 0 (€0 income) to 7 (€7,000 or more)
^c^ A score of 3 or more on the depression subscale of the PHQ-4
^d^ A score of 3 or more on the anxiety subscale of the PHQ-4

Supplementary Table 6: Factors associated with prevalence of hazardous drinking among adults in Germany with Berlin coded as North East Germany

|  | | | Adjusted | |
| --- | --- | --- | --- | --- |
|  | | | OR (95% CI) | p |
| Age^a^ | | | 0.83 (0.79, 0.88) | <.001 |
| Sex | | |  |  |
|  | Male* | |  |  |
|  | Female | | 0.26 (0.24, 0.29) | <.001 |
| Marital status | | |  |  |
|  | Not married* | |  |  |
|  | Married | | 0.93 (0.83, 1.04) | .193 |
| Educational qualifications | | |  |  |
|  | Low* | |  |  |
|  | Medium | | 0.92 (0.81, 1.05) | .224 |
|  | High | | 0.93 (0.81, 1.07) | .305 |
| Monthly income (per €1000)^b^ | | | 1.28 (1.19, 1.36) | <.001 |
| Smoking status | | |  |  |
|  | Never or ex-smoker * | |  |  |
|  | Current smoker | | 2.89 (2.60, 3.22) | <.001 |
| Region of Germany | | |  |  |
|  | South West* | |  |  |
|  | North East | | 1.99 (1.70, 2.34) | <.001 |
|  | North West | | 1.69 (1.50, 1.90) | <.001 |
|  | South East | | 2.34 (1.93, 2.84) | <.001 |
| Depression | | |  |  |
|  | No* | |  |  |
|  | Yes^c^ | | 0.96 (0.73, 1.26) | .758 |
| Anxiety | | |  |  |
|  | No* | |  |  |
|  | Yes^d^ | | 1.38 (0.99, 1.91) | .054 |
| Survey wave | | |  |  |
|  | | Jun to Sep* |  |  |
|  | | Oct to Jan | 0.82 (0.72, 0.93) | .001 |
|  | | Feb to May | 0.91 (0.80, 1.03) | .121 |

Complete cases for all variables of interest except depression and anxiety where multiple imputation was used
* Reference level
^a^ Age variable transformed (divided by the standard deviation[=18.43])
^b^ Range from 0 (€0 income) to 7 (€7,000 or more)
^c^ A score of 3 or more on the depression subscale of the PHQ-4
^d^ A score of 3 or more on the anxiety subscale of the PHQ-4

Supplementary Table 7: Factors associated with prevalence of hazardous drinking among adults in Germany (complete cases for all predictor variables including depression and anxiety)

|  | | | Unadjusted | | Adjusted | |
| --- | --- | --- | --- | --- | --- | --- |
|  | | | OR (95% CI) | p | OR (95% CI) | p |
| Age^a^ | | | 0.77 (0.74, 0.81) | <.001 | 0.86 (0.81, 0.91) | <.001 |
| Sex | | |  |  |  |  |
|  | Male* | |  |  |  |  |
|  | Female | | 0.26 (0.23, 0.29) | <.001 | 0.26 (0.23, 0.30) | <.001 |
| Marital status | | |  |  |  |  |
|  | Not married* | |  |  |  |  |
|  | Married | | 0.81 (0.73, 0.89) | <.001 | 0.94 (0.83, 1.05) | .262 |
| Educational qualifications | | |  |  |  |  |
|  | Low* | |  |  |  |  |
|  | Medium | | 1.14 (1.03, 1.27) | .012 | 1.02 (0.89, 1.17) | .804 |
|  | High | | 1.06 (0.95, 1.19) | .273 | 0.94 (0.81, 1.10) | .437 |
| Monthly income (per €1000)^b^ | | | 1.21 (1.14, 1.29) | <.001 | 1.27 (1.19, 1.37) | <.001 |
| Smoking status | | |  |  |  |  |
|  | Never or ex-smoker* | |  |  |  |  |
|  | Current smoker | | 3.28 (2.96, 3.64) | <.001 | 2.90 (2.58, 3.24) | <.001 |
| Federal state | | |  |  |  |  |
|  | North Rhine-Westphalia* | |  |  |  |  |
|  | Baden-Wuerttemberg | | 0.61 (0.52, 0.73) | <.001 | - | - |
|  | Bavaria | | 0.58 (0.50, 0.69) | <.001 | - | - |
|  | Hesse | | 0.55 (0.44, 0.69) | <.001 | - | - |
|  | Rhineland-Palatinate | | 1.17 (0.92, 1.47) | .199 | - | - |
|  | Saarland | | 0.35 (0.22, 0.57) | <.001 | - | - |
|  | Brandenburg | | 1.49 (1.10, 2.02) | .011 | - | - |
|  | Mecklenburg-Western Pomerania | | 0.91 (0.67, 1.24) | .556 | - | - |
|  | Saxony-Anhalt | | 0.81 (0.58, 1.13) | .221 | - | - |
|  | Berlin | | 2.14 (1.78, 2.57) | <.001 | - | - |
|  | Bremen | | 2.22 (1.43, 3.45) | <.001 | - | - |
|  | Hamburg | | 0.52 (0.35, 0.77) | .001 | - | - |
|  | Lower Saxony | | 0.92 (0.76, 1.10) | .359 | - | - |
|  | Schleswig-Holstein | | 1.39 (1.10, 1.76) | .006 | - | - |
|  | Saxony | | 1.26 (1.00, 1.59) | .048 | - | - |
|  | Thuringia | | 1.21 (0.94, 1.56) | .135 | - | - |
| Region of Germany | | |  |  |  |  |
|  | South West* | | - | - |  |  |
|  | North East | | - | - | 1.69 (1.36, 2.11) | <.001 |
|  | North West | | - | - | 1.90 (1.68, 2.15) | <.001 |
|  | South East | | - | - | 2.17 (1.76, 2.67) | <.001 |
| Depression | | |  |  |  |  |
|  | No* | |  |  |  |  |
|  | Yes^c^ | | 1.00 (0.81, 1.25) | .995 | 0.98 (0.74, 1.28) | .868 |
| Anxiety | | |  |  |  |  |
|  | No* | |  |  |  |  |
|  | Yes^d^ | | 1.17 (0.91, 1.52) | .221 | 1.31 (0.95, 1.81) | .104 |
| Survey wave | | |  |  |  |  |
|  | | Jun to Sep* |  |  |  |  |
|  | | Oct to Jan | 0.91 (0.82, 1.01) | .079 | 0.85 (0.74, 0.96) | .012 |
|  | | Feb to May | 1.01 (0.90, 1.12) | .933 | 0.92 (0.81, 1.05) | .206 |

Complete cases for all variables of interest including depression and anxiety
* Reference level
^a^ Age variable transformed (divided by the standard deviation[=18.43])
^b^ Range from 0 (€0 income) to 7 (€7,000 or more)
^c^ A score of 3 or more on the depression subscale of the PHQ-4
^d^ A score of 3 or more on the anxiety subscale of the PHQ-4

Supplementary Table 8: Ever-drinker and hazardous drinker prevalence estimates, and mean AUDIT-C score and weekly alcohol consumption among youth (aged 14-17 years old) in Germany and stratified by region

|  | Prevalence of ever-drinkers, % (95% CI) | Prevalence of hazardous drinkers, % (95% CI) | AUDIT-C score, mean (SD) | Weekly alcohol consumption in grams, mean (SD) |
| --- | --- | --- | --- | --- |
| Germany (n=352) | 45.4 (40.2, 50.6) | 6.8 (4.2, 9.5) | 1.3 (1.92) | 13.8 (29.64) |
| South West (n=150) | 37.8 (29.9, 45.6) | 4.1 (0.9, 7.3) | 0.8 (1.37) | 9.1 (20.31) |
| North East (n=24) | 82.5 (66.2, 98.8) | 0 | 2.4 (1.64) | 22.7 (16.61) |
| North West (n=155) | 44.8 (36.9, 52.8) | 9.1 (4.5, 13.6) | 1.4 (1.95) | 12.2 (17.51) |
| South East (n=23) | 59.5 (37.8, 81.2) | 16.3 (-0.1, 32.6) | 2.4 (3.46) | 46.1 (86.38) |

Data weighted to be representative of the German population
